# Assessing the Goodness of Fit of Phylogenetic Comparative Methods: A Meta-Analysis and Simulation Study

**DOI:** 10.1371/journal.pone.0067001

**Published:** 2013-06-27

**Authors:** Dwueng-Chwuan Jhwueng

**Affiliations:** Department of Statistics, Feng-Chia University, Taichung, Taiwan, Republic of China; University of California, San Diego, United States of America

## Abstract

**Background:**

Phylogenetic comparative methods (PCMs) have been applied widely in analyzing data from related species but their fit to data is rarely assessed.

**Question:**

Can one determine whether any particular comparative method is typically more appropriate than others by examining comparative data sets?

**Data:**

I conducted a meta-analysis of 122 phylogenetic data sets found by searching all papers in JEB, Blackwell Synergy and JSTOR published in 2002–2005 for the purpose of assessing the fit of PCMs. The number of species in these data sets ranged from 9 to 117.

**Analysis Method:**

I used the Akaike information criterion to compare PCMs, and then fit PCMs to bivariate data sets through REML analysis. Correlation estimates between two traits and bootstrapped confidence intervals of correlations from each model were also compared.

**Conclusions:**

For phylogenies of less than one hundred taxa, the Independent Contrast method and the independent, non-phylogenetic models provide the best fit.For bivariate analysis, correlations from different PCMs are qualitatively similar so that actual correlations from real data seem to be robust to the PCM chosen for the analysis. Therefore, researchers might apply the PCM they believe best describes the evolutionary mechanisms underlying their data.

## Introduction

Over the past 20 years, many methods have been developed for incorporating phylogenies in comparative analysis. One of the most popular methods was proposed by Felsenstein and is known as the Felsenstein Independent Contrasts method (FIC), which assumes that trait values change according to the Brownian-motion process [1].

Cheverud et al. applied a general network autocorrelation model (PA) to phylogenetic comparative analysis, dividing the trait value (T) into an inherited phylogenetic value (P) and an independent specific value (S) [2] (see also [3]). Martins and Hansen proposed a phylogenetic generalized least square model (PGLS) assuming an Ornstein-Uhlenbeck (OU) process for the evolutionary change along the phylogeny. Such a model imagines that there is a rubber-band like process drawing extreme values back towards a common optimum mean value for the trait [4]. More recent work continues to develop this OU model [5], [6]. The phylogenetic mixed model (PMM) was first proposed by Lynch [7] and clarified by Housworth et al. for a single trait [8], and in the univariate case is identical to Pagel's lambda method [9]. The model allows for one component of the trait to follow a Brownian motion process along the phylogeny and a second component to be independent of the phylogeny. The model estimates how much of the trait is due to each component.

The purpose of this article is to compare the fit of these various methods for incorporating the phylogeny to comparative data found in the literature. Can one tell, by examining data typically collected for a comparative analysis, that one comparative method is decidedly more appropriate than another? Furthermore, comparative methods are most commonly used to examine two or more traits measured on the same set of species, but not all comparative methods have had their bivariate analogs delineated. I describe the appropriate bivariate variance-covariance structure for each model, some of which were previously unknown. I consider the use of PCMs in bivariate analyses where the parameter of interest is the correlation between two traits for a group of species, with the goal of determining the effect of model choice on the estimates of correlations. Are the correlation estimates qualitatively concordant (having the same sign) or do the methods give wildly different correlation estimates for given real data sets?

Modifications are required to three of these methods in order for the AIC comparisons to be valid [10]. I make a trivial modification to FIC to get maximum likelihood rather than restricted maximum likelihood estimates. I make a more substantial modification to the autocorrelation model by not normalizing the data in advance of determining its error structure. As the OU process should recover the Brownian motion process when the constraint parameter tends to zero, I make a modification to the PGLS as given in [4] so that this property is preserved. This same modification was used in [5].

## Methods and Materials

### Data Selection

I searched for published phylogenetic data sets in JEB, Blackwell Synergy and JSTOR using the keywords: ((Comparative methods OR Comparative analysis) AND independent contrasts) for 2002–2005. I included only articles that contained a phylogeny and the raw data for continuously distributed traits. All data sets contained averaged trait values; some also provided sample sizes and standard deviations or standard errors. These criteria yielded 43 articles, which were pruned further by eliminating studies where the species were from more than one order (so as to increase the chance that the species experienced a single model of evolution). Note that the choice of order as the cut-off is arbitrary; other authors have used other cutoffs such as families [11] and genera [5]. The final assemblage of data sets included 122 traits (some papers had multiple traits) and 47 phylogenetic trees. Data set size ranged from 9 to 117 species. The flow of information through the different phases of a systematic review is reported in Figure S1 in supporting information section. The references for the data sets are listed in Table [11]–[34].

**Table 1:** References for the meta analysis.

References	Size	Species
[11]	20	Centrarchid fishes
[12]	17	Tropical Savannah bat
[13]	19	Tribe collinsieae
[14]	37	Tinamous
[15]	24	Lizards
[16]	34	Lizards
[17]	11	Macaranga ant-plants
[18]	27	Ungulates macropods
[19]	10	Drosophila
[20]	29	Pine
[21]	40	Parasitoid wasps
[22]	14	Notothenioid fishes
[23]	12	Gobies
[24]	14	Snow skinks
[25]	17	Anurans
[26]	20	Drosophila
[27]	17	Lizard
[28]	22	Neotropical marsupials
[29]	117	Diurnal raptors
[30]	21	Cnidaria
[31]	18	Ungulates
[32]	37	Woodcreepers
[33]	9	Lizards
[34]	42	Figs

Phylogenies and comparative data are collected from the literature. The sample sizes (the number of the taxa) are shown is the second column.

### The Phylogenetic Similarity Matrix *G*

PCM approaches typically require a phylogeny with branch lengths. To recover this, I used a ruler to measure the branch lengths. Some of the phylogenies were chronograms, where branch length is proportional to time, and others were cladograms; the former would be expected to have better branch lengths for PCM. I converted each phylogeny into a similarity matrix ***G***. The diagonal elements of ***G***, 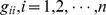, represent the correlation of a species with itself and so equal 1.0. The off-diagonal elements 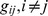, represent the relative evolutionary time shared by two species 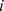 and 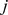 so that 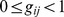 when 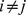. Figure  shows a rooted phylogeny of 5 taxa with the assigned branch lengths.

**Figure 1:**
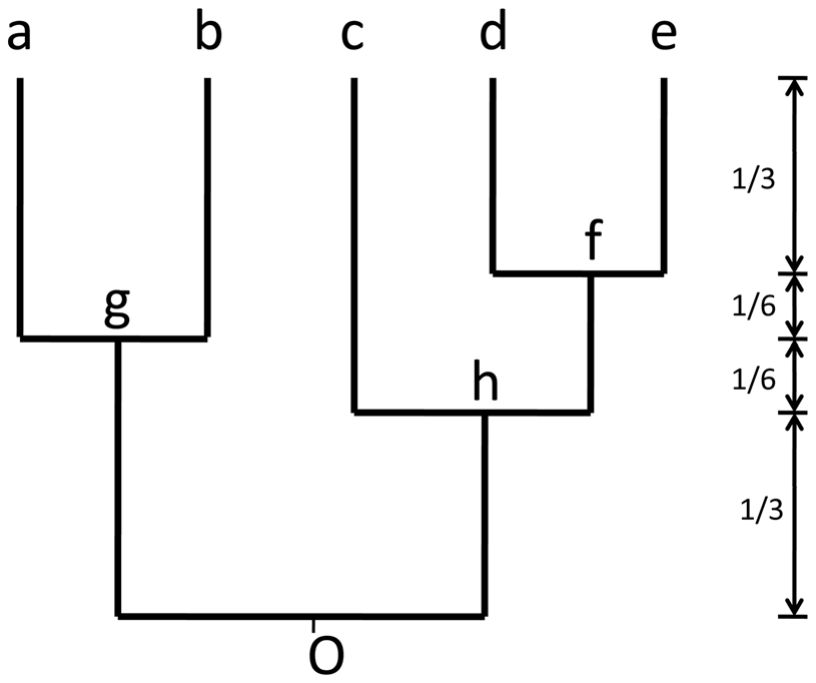
A rooted phylogenetic tree of 5 taxa. Tips a, b, c, d, e; interior nodes f, g, h and the root O.

The associated similarity matrix ***G*** is
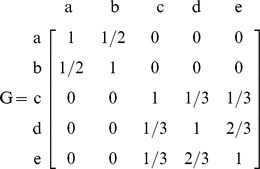



### Statistical Model and Statistical Fit by MLE Estimation

#### Univariate Model

Given the assumption of a Markov process of trait evolution, trait values 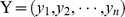 are assumed to follow the multivariate normal distribution
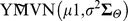



with the overall mean 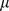
and variance 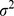. 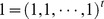 is the vector of ones.

Each PCM results in a different variance-covariance structure 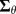, for the data:

ID 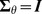, the identity matrix.

FIC 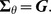


PMM 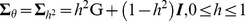.

PA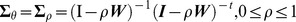.

OU 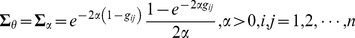.

Several of the PCMs have a free parameter (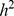 for PMM, 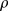 for PA, 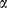 for OU); for simplicity, I refer to this parameter as 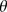. Note that for FIC, I make a trivial modification to get maximum likelihood rather than restricted maximum likelihood estimates. For PA, ***W*** is the connectivity matrix [2]. For OU, 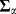 is identical to the covariance structure in [5].

The negative log likelihood function is
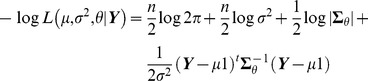
where 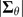 is the determinant of 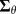.

The MLEs for the mean and variance are the function of 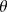
where


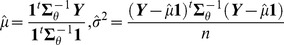
, respectively.

The MLE estimator 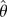 is obtained by optimizing the negative log likelihood function 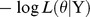 on the domains: 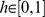 for PMM, 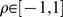 for PA, and 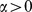 for OU, respectively.

#### Bivariate Model

The bivariate model for traits ***X*** and ***Y*** measured at time *t* has a general form. Let 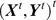 follow a multivariate normal distribution with mean 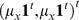 and a *2n* by *2n* covariance matrix 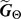 where 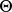 represents the model-specific parameters in the variance-covariance structure. The statistical model is
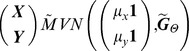




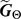
 for each model is

ID 
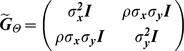
.

FIC 
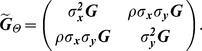



PMM
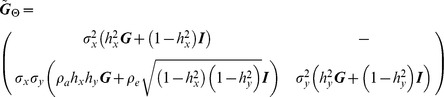
where the correlation between traits ***X*** and ***Y*** is



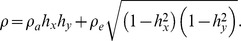



PA 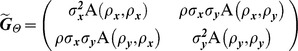



where 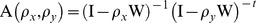.

OU 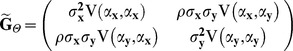



where 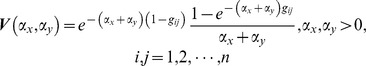.

The negative log likelihood function is
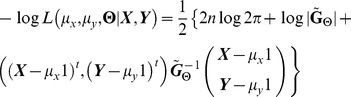



where 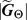 is the determinant of 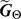. I use restricted maximum likelihood methods (REML) to estimate the correlation between two traits, as adjusted by the effect of the phylogeny. REML eliminates the need to estimate means and often produces less bias in the variances and correlation estimators. I used Powell's method to optimize the maximum likelihood estimators (after reducing the dimension of the search by solving for some parameters in terms of others; details available upon request). This method uses one-dimensional line searches in increasingly independent best directions, while periodically resetting the directions to be orthogonal. It is fast and efficient when the function is quadratic or close to quadratic, as likelihood functions often are close to their maximum [35]–[36]. There is no published maximum likelihood method for bivariate PA and OU. To create one, I propose the appropriate variance-covariance structure for these two methods:

PA:

The univariate phylogenetic autoregressive model proposed by Cheverud et al. [2] adapted the spatial autocorrelation model in [37]. The modified model I propose for univariate data analysis is
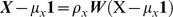
where 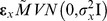 and ***W*** is the phylogenetic connectivity matrix with zeros on the diagonal and rows that sum to one. The autocorrelation coefficient, 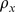, measures the impact of the phylogenetic effect on the traits. The residual 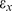 is independent of 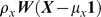 and can be regarded as the value gained or loss due to non-phylogenetic component.

Transforming the equation, we have
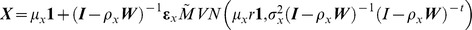



For bivariate analysis, let 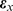 and 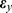 be the residuals for the traits ***X*** and ***Y***, respectively. As in the univariate case, the residuals 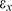 and 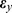 are independent of the phylogenetic components 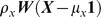 and 
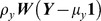
. I now assume the correlation between the two residuals exists and let the correlation equal to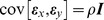.

Then, the covariance between the pair of traits is
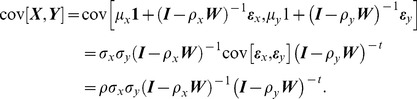



The statistical model for the bivariate phylogenetic autoregressive model is therefore,
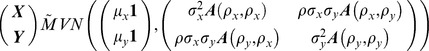
where 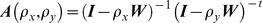.

OU:

Martins and Hansen considered species evolving under the OU process where a selection force pulls the trait back to an optimum. Thus, the OU process can be used to model stabilizing selection [4]. The univariate OU model assumes that the trait at time *t*, 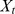, satisfies
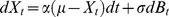
Where 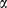 measures the magnitude selection force, 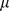 is the optimum of the trait, and 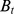 is Brownian motion. The selection force acts strongly towards the optimum when the trait is far from the optimum and weakly if the trait is close to the optimum. For the bivariate analog, there are two constraining force parameters, 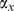 for trait ***X*** and 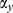 for trait ***Y***.

Assuming that the constraining force parameters 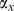 and 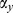 are constants during the evolutionary process, I propose the bivariate statistical model
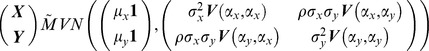
where 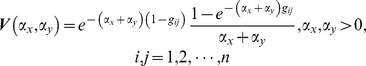.

The covariance structure is developed by the following mathematical property:

#### Theorem 1

**Figure 2:**
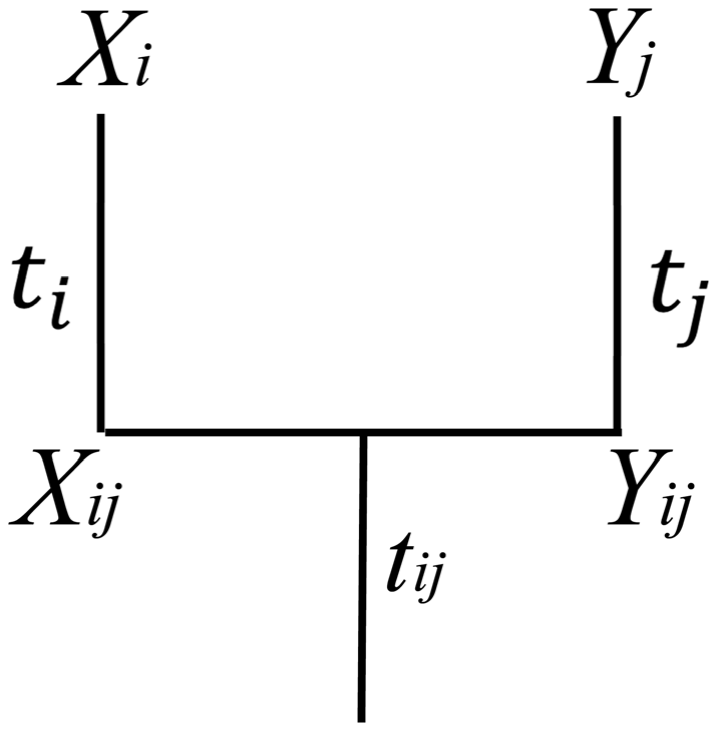
A two taxon phylogenetic tree of two species *i* and *j* with trait values 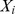
 **and** 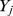. 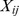 and 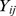 are the trait values for the common ancestor of *i* and *j*. 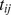 measures the branch length from the root to the most common ancestor of species *i* and *j*. 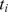,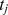 are the branch length for species *i* and *j* since they diverged.

Let 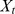 and 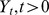 be two OU process random variables. Given a rooted phylogeny for trait evolution, assuming that the constraining force parameters 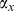 and 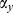 are constants during the evolutionary process, the covariance between the trait 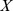 of species *i* and the trait 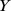 of species *j* is
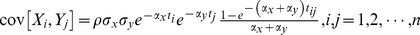
where 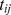 measures the branch length from the root to the most common ancestor of species *i* and *j*; 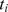, 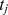 are the branch lengths for species *i* and *j* since they diverged

(Figure 2). Proof of **Theorem 1** is provided in appendix S1.

### Model Selection for Univariate Data

For univariate data analysis, the fitted models were compared using the Akaike Information Criteria (AIC), in order to measure fit to data of a model [10].
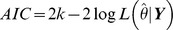
where 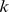 is the number of parameters, 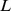 is the likelihood function, and 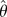 is the MLE estimator(s) in that model.

Hurvich and Tsai found AIC could over-fit models and be biased if there are too many parameters in comparison to the sample size. They proposed a modification of AIC when the ratio of sample size to the number of parameter does not exceed 40 (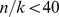), AICc [38]:
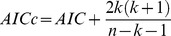



Since 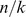 in this study is always less than 40, I used AICc for model selection.

Another popular model selection method, Bayesian Information criterion (BIC) where 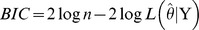, is based on an asymptotic result derived under the assumptions that the data distribution is in the exponential family [39]. BIC is not appropriate in model selection for biological phenotypic data sets because, when sample size is small, BIC tends to select unfitted models with large bias, which results in difficulty in inference [40].

### Simulation

#### Do Some Models really fit better than others?

For a given dataset, one can get a ranked list of models, including the best model, by getting point estimates of AICc differences. However, it is also useful to get the confidence intervals for these differences. Thus, after determining which model fits a given data set best, I proceeded to test whether it fits significantly better than the other models by simulating the distribution of AICc differences, using a procedure suggested in [40]. Given a set of models indexed by 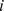, define 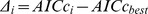 where 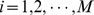 is the model index and the term *best* is the index of the best model. In this study, 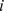 represents ID, FIC, PMM, PA, or OU. I treated 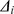 as a random variable and suggested the following bootstrap technique for determining a confidence interval for 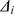. If FIC is the best model for the data ***Y***, generate new bootstrap samples 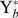 from FIC using the MLE from the original data. For each sample, 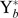, determine the AICc values for each model. Let 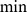 be the index of the model with smallest AICc value for the bootstrapped data. Define the random variable 
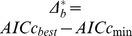
. I then order the 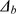 in increasing order for the 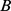 bootstrapping samples. Re-using the index 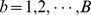 but for the ordered values, the 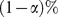 confidence set for 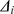 is CI  =  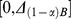. If 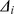 (the original difference for model *i* versus the best model) falls outside this confidence interval, the null hypothesis that the two models fit the data equally well is rejected and I conclude that the best selected model under AICc is significantly better than model *i*. I set 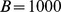 to get a reliable result on the upper tail of 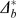.

#### Robustness

The comparative data sets usually consist of mean trait values derived from measuring a finite number of samples, which are subject to both measurement and sampling error. The phylogeny, even when given as a rooted molecular clock tree, also has uncertainty in branch lengths and topology because phylogenies are often obtained by a sample of DNA sequences from the species involved. Accordingly, I consider the robustness of my model selection results to perturbations of the trait values and the phylogeny. Both procedures are described below.

#### Perturbation of Comparative Data

Consider collecting *m* samples from a particular species yielding trait values 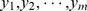. Let *se* be the standard error and let 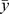 be the sample mean. All of *m*, *se*, and 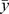 are typically reported in the studies used in this analysis. I perturbed the species mean value by using the formula 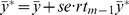
where *rt* is a random sample from the *t* distribution with 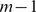 degrees of freedom. This is a reasonable approach because the sample mean will be approximately *t*-distributed in most cases. However, biological data often follow a log normal distribution with a natural bound of zero and with no specific upper bound. On occasion, when the sample size is not large and the underlying data are log normal, using 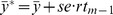 to generate 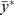
can give impossible (negative) values with a substantial probability. Under such circumstances, I apply an alternative resampling technique aimed at reproducing the log normal data. Note that if 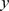 is a log normal random variable, then 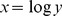 is a normal random variable. The second order Taylor series approximation for the function 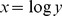 at the point 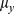 is



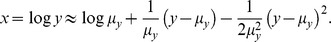



Given the sample mean 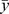 and sample variance 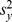 for a log normal variable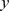, the distribution of the normal variable 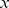 (with mean 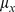
and variance 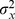) is approximately



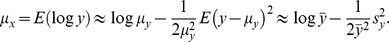



Using the linear part,



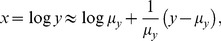
the variance of 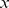 can be approximated as



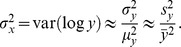



I sampled from the normal distribution with appropriate mean and variance and exponentiated to get a sample from the log normal distribution. For a sample of size *m*, I simulate 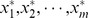 from normal distribution with mean 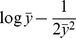 and variance 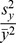 . Then the perturbed data 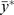 can be obtained as



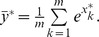



#### Perturbation of Phylogeny

Stone considered the effect of local phylogenetic perturbations on the regression fit. He studied how tree misspecification could influence the phylogenetic regression given a Brownian motion model of evolution. He found that branch length misspecification can be easily explained in terms of the reweighting of contrast scores between subtrees [41]. I used a likelihood-based approach rather than regression to investigate how branch length misspecification could influence the model selection. To do this, I first perturbed the phylogenetic tree by randomly varying the branch lengths without changing the topology. Recall that the phylogenetic tree is scaled so that the length from the root to each tip is one. The unit length is decomposed into 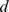 segments with lengths 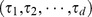 by identifying each 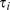 as the time difference between two adjacent nodes in the phylogenetic tree. Thus, 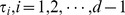 are times between the 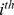
and the 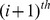 speciation events and 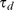 is the time between the tip and the most recent node in the phylogenetic tree. In terms of the entries of the relationship matrix ***G***, let 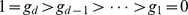 be the ranking of the distinct entries in ***G***, then we have 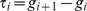. To perturb the branch lengths 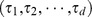 but retain the topology of the original phylogeny, the procedure is described in the following. I first treated 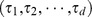 as a 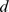-dimensional random variable from a Dirichlet distribution generated by drawing 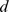 independent random samples, 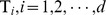, each from a Gamma distribution with rate parameter 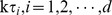 where 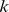 is an arbitrary but positive constant. The desired 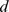
- tuple sample 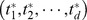 from the Dirichlet distribution with parameter 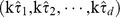 is determined by



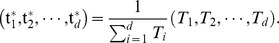



Note that 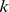 is an arbitrary scaling variable that always preserves the correct mean. That is,



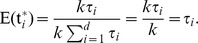



The Dirichlet distribution has a mode given by


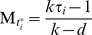
, where 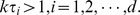


The choice of 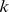 is thus determined by 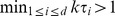
_._

I chose a positive integer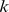,
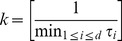
where [*a*] returns the integer closest but less than *a*.

Because the mode 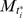 of 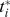 is not equal to the expectation of 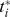, such choice of *k* does not guarantee that the distribution of 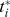 is centered or symmetric around its mean 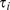. The mode converges to the expected mean when 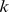 approach to infinity (i.e. 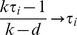 as 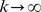). However, although choosing larger 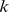 helps to center the distribution around 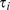, picking 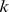 too large will cause the samples 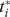 to be tightly centered around the given estimate 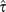. My choice of 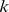 is designed to be the minimal needed to prevent the phylogenetic tree from varying too wildly from the given one while still adequately testing robustness.

### PCMs Comparison by Confidence Intervals for the Correlation

For bivariate data analysis, I generated the confidence interval by creating bivariate samples 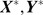 using the MLE estimators of all the model parameters and re-estimating the correlation. I created 1000 pairs of samples 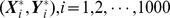 and performed the REML analysis to obtain the MLEs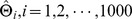. The 95 % confidence interval (CI) for the correlation under the hypothesis testing is constructed from the ordered MLEs for the correlation 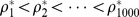 with the cut off CI  = 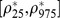. The correlation is significantly positive at the 5% level if 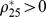, significantly negative at the 5% level if 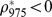 and otherwise not significantly different from zero at the 5% level.

## Results

### Model Selection under AICc

I report the summary of the results from the simulation study in Table 2. By the first row of Table 2, most of the data sets are best described by either of the simplest models: the independent model (ID) or by Brownian motion (FIC). The entire table shows the performance of model-fitting of each model when competing with other models. If the best fit model is independent (no phylogenetic effect), then that model usually fits significantly better than other models. However, if the model that best fits the data is Brownian motion (FIC), then other models, except phylogenetic autocorrelation (PA), have a substantial probability of fitting the data as well. For other parameter-rich models, PMM and OU usually fit significantly better than other models; however, they do not fit statistically significant better than each other. PA fits significantly better than other models except for the independent (no phylogenetic model).

**Table 2:** Summary of the data sets that fit multiple models.

	IDb	FICb	PMMb	PAb	OUb
	(*n* = 36)	(*n* = 67)	(*n* = 4)	(*n* = 5)	(*n* = 10)
ID	-	0.48	1	0	0.8
FIC	0.86	-	0.75	0.8	0.7
PMM	0.94	0.06	-	0.8	0.4
PA	0.89	0.81	1	-	0.9
OU	0.86	0.12	0.5	0.8	-

In the first row of the table, *n* represents the number of data sets that best fit a particular model under AICc. In each column, the entries represent the ratio for which the best fit model fits significantly better than other models (see “Do some models really fit better than others?”). For instance, the second column shows that among 36 data sets that ID best fits the data, the ratio that ID fits significantly better than FIC, PMM, PA and OU are 0.86, 0.94, 0.89 and 0.86, respectively.

### Robustness for Models under Perturbing Data and Phylogeny

**Figure 3:**
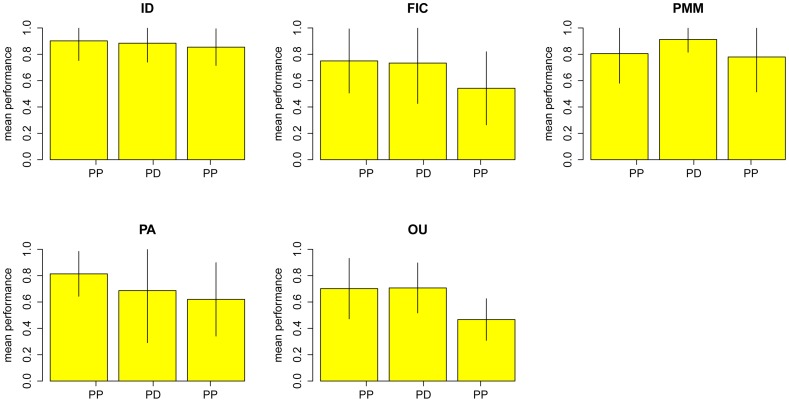
Summary of the sensitivity analysis for perturbing comparative data and phylogeny. The height for each bar graph represents the mean performance of the model where the vertical line on each bar graph represents the standard deviation of the performance. PP: Perturbing the phylogeny; PD: Perturbing the data only; PB: Perturbing both phylogeny and data.

There were 64 traits for which standard error and sample size were reported. For each trait, I simulate one thousand perturbed data sets and phylogenies. The performance of the best model for a trait is evaluated by proportion of simulated data sets on which that model achieves the best fit. The mean performance is the average value of the performances across studies. The results of the perturbation analyses are shown in Figure 3. Data sets that best fit by the independent model are the most robust to perturbations while those whose best fit is a Brownian motion model (FIC) are less robust. Data sets that are best fit by the Brownian motion model (FIC), PMM, and OU seem to be more sensitive to perturbations in the phylogeny than to perturbations in the comparative data themselves.

### Model Adequacy for PCMs

I also evaluated model adequacy for PCMs. The purpose is to investigate how well the model describes the underlying process that generated the trait data. Essentially, if a model simulates datasets that are indistinguishable from the observed datasets, the model adequately describes the data. I first simulated a thousand datasets by parametric bootstrapping using the MLE obtained from the empirical data under each model and then re-evaluated the likelihood from the simulated data for each model. The model is considered inadequate if the log likelihood for the empirical data falls out of the 95% confidence interval from the simulated data.

**Figure 4:**
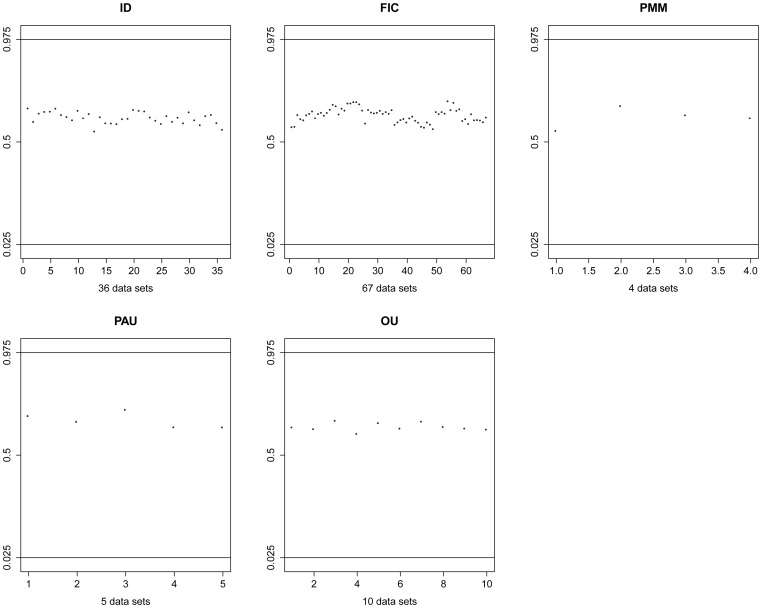
Summary of testing model adequacy. In each plot the 95 % confidence interval is shown by two horizontal lines (0.025 and 0.975). Each point in the plot represents the rank in the 95 % confidence interval for the empirical data sets. In no cases are the empirical data sets outliers relative to the simulated datasets, suggesting model adequacy.

Figure 4 summarizes the result where scatter plots are shown to examine the adequacy of the AIC best model. The likelihood for the empirical datasets falls well within the 95% confidence interval for the simulated data from each model. Expressed as a percentile of the simulated scores, the empirical data averaged in the 61^st^ percentile for ID, 62^nd^ percentile for BM, 61^st^ percentile for PMM, 66^th^ percentile for PAU, and 63^rd^ percentile for OU.

### Assessing Goodness of Fit for PCMs

It might be the model for fitting data is over fitted. To investigate this, I simulate data using the true MLE under the best model. Then the fit of other models is then evaluated.

**Figure 5:**
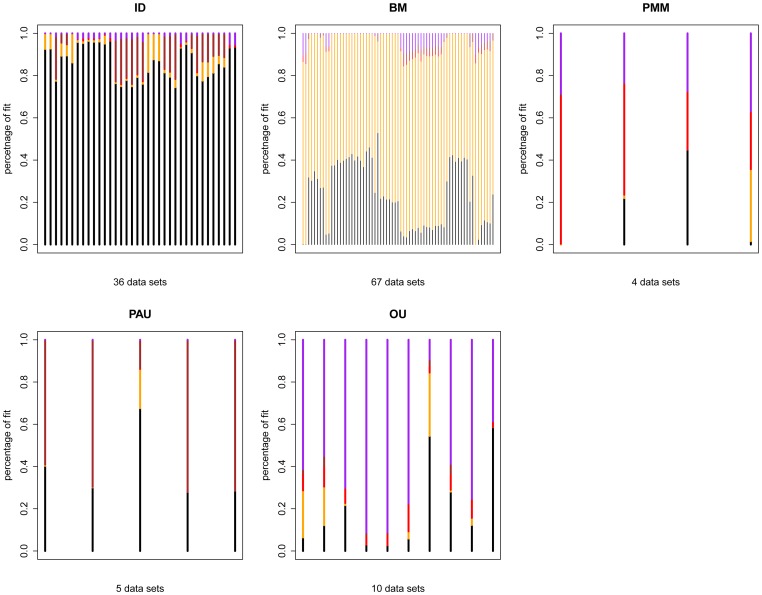
Investigating over fitting issue for PCMs using the trait data from literature. Each vertical line with different color in the plot represents the ratio of the fit: ID (black), BM (orange), PMM (red), PAU (brown), OU (purple).

The result is shown in Table 3 and Figure 5. The diagonal entries in Table 3 show the average ratio of fit for the best model. Datasets simulated under ID have ID as the best fitting model 86% of the time, FIC has the best fit 72% of time. For more complicated models, datasets simulated under PMM have PMM as the best fitting model 45% of the time, PA has the best fit 57 % of time and OU has the best fit of 63 % of time which encounter over fitting issue. For PMM, as the combination model of ID and FIC, could be over fitted while the heritability parameter close to 0 or 1. In this case, ID and FIC would be the better fit. Similarly PA is an extended model for ID where the zero autocorrelation is detected.

**Table 3:** Average fit for model fitting.

	IDb	FICb	PMMb	PAb	OUb
	(*n* = 36)	(*n* = 67)	(n = 4)	(*n* = 5)	(*n* = 10)
ID	0.86	0.24	0.17	0.39	0.21
FIC	0.04	0.72	0.09	0.04	0.08
PMM	0.00	0.01	0.45	0.00	0.07
PA	0.08	0.01	0.57	0.57	0.02
OU	0.01	0.03	0.00	0.00	0.63

In the first row of the table, *n* represents the number of studies that best fit a particular model under AICc. In each column, the entries represent the average ratio for the best model across the studies.

### Comparing Correlation Estimates from the PCMs

**Figure 6:**
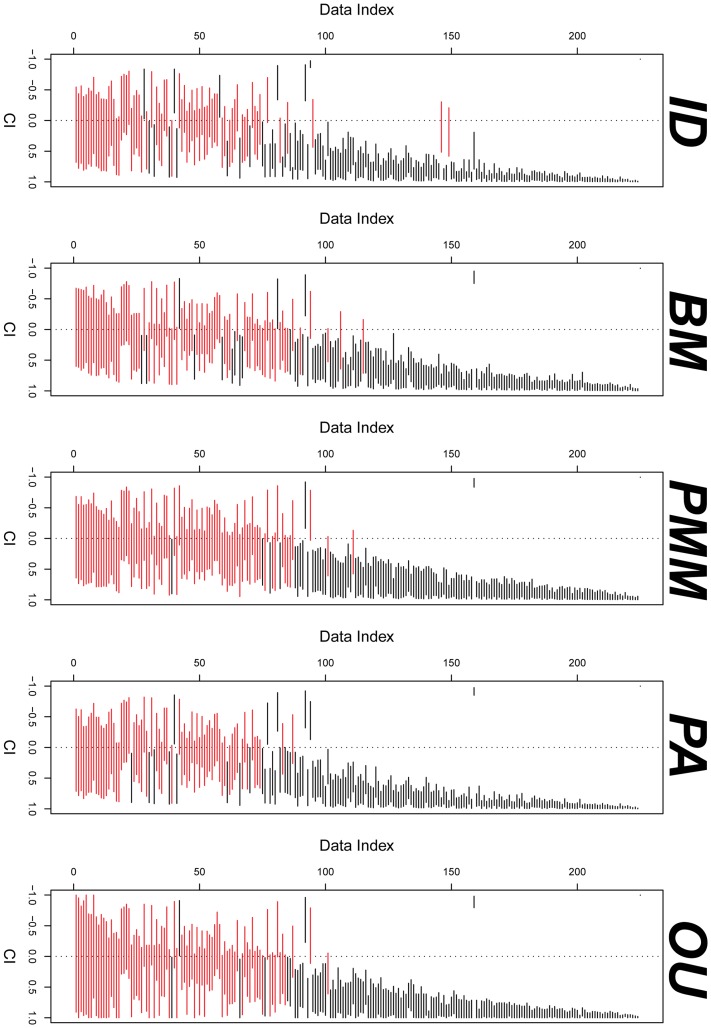
The confidence intervals of correlation for PCMs. Each line represents a confidence interval for a data set. Red lines include zero correlation in the confidence intervals.

I analyzed 225 bivariate data sets with 23 phylogenies. Figure 6 gives the comparison of the confidence intervals for correlations. Correlations tend to be either positive or include zero in their confidence intervals.

**Figure 7:**
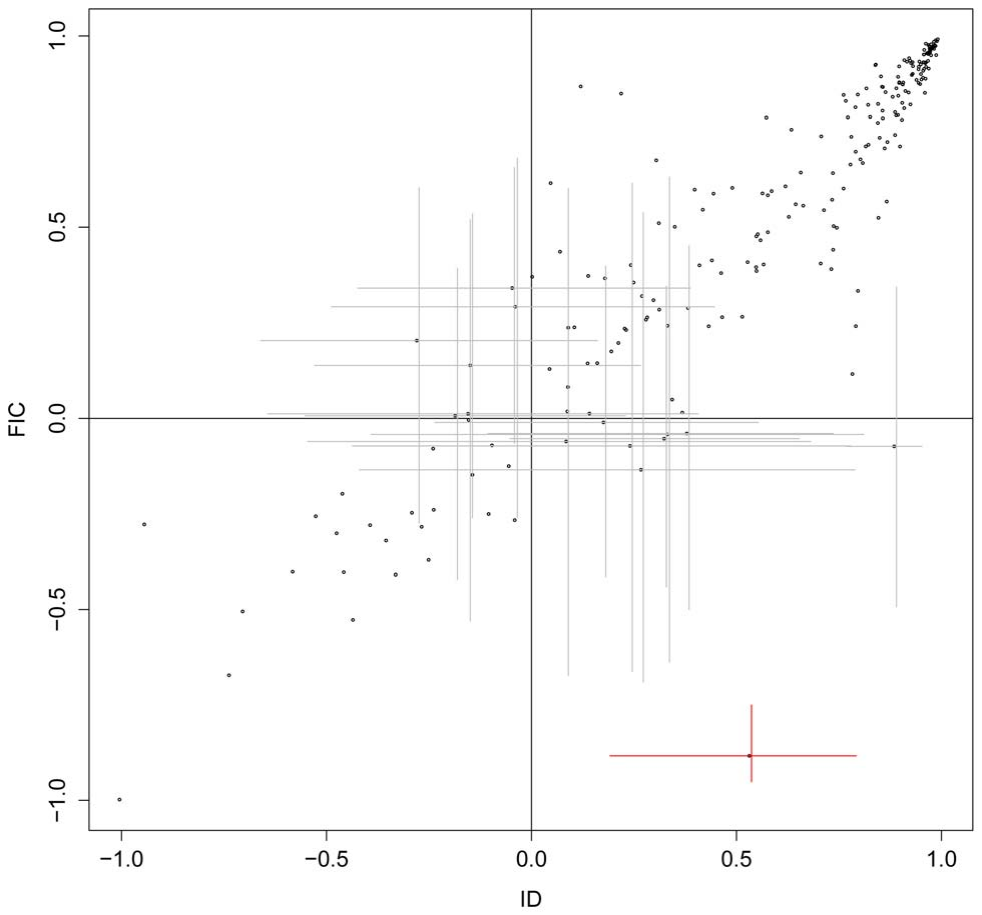
Comparison of correlation between ID and FIC. On the plots, points in the second and the fourth quadrants are the correlations estimates estimated discordantly (different sign) by ID and FIC. Those points are then shown with the confidence interval. Only such one point, indicated in red, has confidence intervals for both correlations excluding zero, suggesting a difference in sign of the correlation that cannot be due just to uncertainty in the correlation estimates.

Figure 7 shows the comparison of correlation between ID and FIC. Most (93.4%) correlations have the same sign. There are only fourteen data sets for which there are estimated correlations of opposite signs that show the disagreement (the points in the second and the fourth quadrant) and for only one of these do the confidence intervals not include areas of agreement.

**Figure 8:**
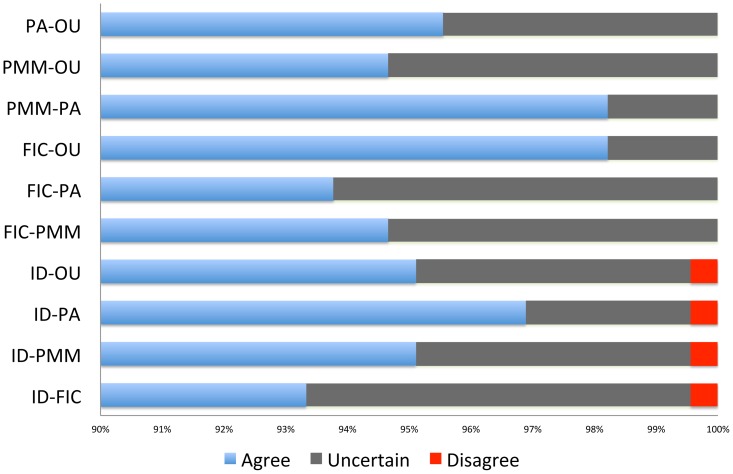
Concordance of the correlation estimates between PCMs. Blue bars indicates agreement in sign in correlation. Dark grey bars indicate disagreement of sign for the point estimates of the correlation, but with agreement possible given the confidence intervals on the estimates. Red bars indicate conflict in sign that cannot be reconciled by accounting for uncertainty.

Figure 8 gives the summary of the concordance of the correlation estimates between PCMs by comparing the confidence intervals. Figure 6, Figure 7, and Figure 8 suggest that most estimated correlations are concordant. Thus, if there were a significant positive (or negative) correlation under one model, using a different model would also yield a significant positive (or negative) correlation. This is a very reassuring result for the use of PCMs. Similar results have been found, but for fewer models, by previous authors [42]–[43].

## Discussion

Harmon et al. compared BM (Brownian motion), SSP (single stationary peak), and EB (early burst) models of morphological evolution and found little support for the EB model, whereas both other models, particularly that of SSP, were commonly supported [44]. In this work, I have looked at the most common bivariate models, but also created univariate versions of them to see how well they explain evolution of single traits: one expects that models that do not work well in the univariate case would also not perform well in the multivariate case, though this has not been formally shown.

I have shown that ID and FIC are most frequently chosen as the best model under the univariate analysis. This is in part due to the penalty AICc places on models with more parameters. If all else is equal (including the likelihood), then the AICc differences for a pair of models with one having one more parameter is 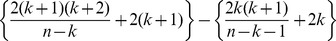. For two versus three parameters, the difference is 
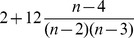
 where 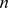
is the sample size. The number of species in the data sets under consideration in this study ranged from 9 to 117 and so this penalty ranged from 2.10 to 3.43. Some of these models are special cases of others. For instance, when the heritability parameter *h* in the PMM model converges to 0 or 1, PMM is identical to ID or FIC, and thus the likelihood for PMM and one of the extreme models would be identical in this case. However, AICc penalizes PMM for using an additional parameter. This also occurs for PA: when the autocorrelation 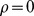, PA is identical to ID. Similarly OU is identical to FIC when 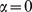. In these cases, simpler models (ID and FIC) provide a better fit for those data.

In the univariate case, it appears that when the best-fitting model is ID, other models will almost always fit more poorly. However, when the best model is slightly more complex, like FIC, it is usually hard to reject other models. This makes sense: FIC (based on likelihood rather than REML) is nested within other models with just one parameter, so it can be hard to distinguish them, especially in studies with moderate to small numbers of taxa. Researchers often just use a point estimate to determine the best model; however, I have shown there is often quite large uncertainty as to the best model. Modifying the phylogeny can result in dramatic shifts in the best model, but modifying the trait data had a much smaller effect. This suggests that when estimating the best model is important, a premium should be placed on estimating the phylogeny well. One caveat, though, is that the uncertainty added to the phylogenies was not based on empirical estimates. However, the magnitude used was fairly minimal (branch lengths differed from original branch lengths by 0.3 % on average with 3.2 % variation across different phylogenies) yet still had a major effect on model choice, suggesting that this may also be important in empirical analyses.

Despite the adequacy of models examined in this analysis, there are many ways in which the true evolutionary process may deviate from the models. For example, differing rates of evolution across taxa [45]; multiple optima [5]; or multiple optima, multiple rate and multiple attractors [6] may occur throughout a clade. Much work in this area uses parametric approaches, but non-parametric approaches may hold promise. Development of corresponding bivariate models may aid the exploration of correlation and appropriate alternative summaries of association in comparative analyses of multiple traits.

The results obtained here suggest caution when applying bivariate PCMs. In over half the cases, different PCMs give the same sign of correlation but in about a quarter of the cases, different methods resulted in correlations with different signs. Different methods gave non-overlapping confidence intervals for correlations in only a small proportion of cases. Price found that FIC and ID gave correlations with different signs in 14.7 % of cases [42], whereas I found this happened in 6.6 % of cases for those models. Such discrepancies occur with real data. For example, Fig. 3B and Fig. 3E in [46] showed an example in which leaf life span and leaf size in angiosperms and conifers had a negative correlation when using the ID and a positive relationship when using FIC; for most other traits, the sign of the correlation was the same regardless of model.

One surprising result is that all pairs of PCM agreed and disagreed by about the same amount, regardless of whether they were fairly similar models (FIC-PA) or very different (FIC-ID). Note that I have analyzed various univariate models in the same framework so as to allow model selection; this has yet to be done for the multivariate case. Revell has shown that one cannot just take univariate model selection results to infer the best multivariate model [47]. Thus, developing model selection approaches for the variety of available multivariate models remains important work for the future.

## Supporting Information

Appendix S1 **Proof of Theorem 1.**
(PDF)

Figure S1 **Flow of information through the different phases of a systematic review.**
(DOCX)

Table S1 **Checklist of items to include when reporting a systematic review or meta-analysis.**
(DOCX)
