# Correction: Assessing the Goodness of Fit of Phylogenetic Comparative Methods: A Meta-Analysis and Simulation Study

**DOI:** 10.1371/annotation/7b304b36-72c3-40b5-8154-2848315fd5d4

**Published:** 2014-01-06

**Authors:** Dwueng-Chwuan Jhwueng

In the Methods section, there was an error in the BIC formula located in the section titled "Model Selection for Univariate Data". It should read BIC = k log n - 2 log L(theta_hat |Y). Please view the complete, correct equation here: http://plosone.org/corrections/pone.0067001.eBIC.cn.tif

