# Correction: Assessing the Goodness of Fit of Phylogenetic Comparative Methods: A Meta-Analysis and Simulation Study

**DOI:** 10.1371/journal.pone.0315967

**Published:** 2024-12-12

**Authors:** The PLOS One Staff

The tilde (~) symbol is incorrectly formatted throughout the article. It should be separated from the text. Hence, the equation in Statistical Model and Statistical Fit by MLE Estimation subsection of Methods and Materials should appear as Y~MVN and also in other equations where (~) is present.

The publisher apologizes for the error.
