# Supplementary material for: Assessing the Goodness of Fit of Phylogenetic Comparative Methods: A Meta-Analysis and Simulation Study
Source: PLoS One. 2013 Jun 27;8(6):e67001. doi: 10.1371/journal.pone.0067001 (PMC3694968; doi:10.1371/journal.pone.0067001)
Supplement: Appendix S1 — Proof of Theorem 1. (PDF) [file pone.0067001.s001.pdf]

Appendix S1

Theorem 1: Let X_t and $Y_t,\ t > 0$ be two OU process random variables. Given a rooted phylogeny for trait evolution, assuming that the constraining force parameters α_x and α_y are constants during the evolutionary process, the covariance between the trait *X* of species *i* and the trait *Y* of species *j* is

$$cov[X_i, Y_j] = \rho\sigma_x\sigma_y e^{-\alpha_x t_i} e^{-\alpha_y t_j} \frac{1-e^{-(\alpha_x+\alpha_y)t_{ij}}}{\alpha_x+\alpha_y}, i, j = 1,2,\cdots,n.$$

where t_{ij} measures the branch length from the root to the most common ancestor of species *i* and *j*; t_i,t_j are the branch lengths for species *i* and *j* since they diverged.

Proof:

As the covariation between two traits does not depend on their means, it suffices to consider the system of non mean-reverting SDEs.

$$dX_t = -\alpha_x X_t dt + \sigma_x dB_{x,t}$$

$$dY_t = -\alpha_y Y_t dt + \sigma_y dB_{y,t}$$

Discretize the SDEs, we have

$$X_{t+\Delta t} = (1 - \alpha_x \Delta t)X_t + \sigma_x(B_{x,t+\Delta t} - B_{x,t})$$

$$Y_{t+\Delta t} = (1 - \alpha_y \Delta t)Y_t + \sigma_y(B_{y,t+\Delta t} - B_{y,t})$$

where $\Delta t = \frac{t}{n}$ is the infinitesimal unit of time.

Given a rooted phylogeny, we can decompose the trait values at tip *i* and *j*, X_i, Y_j as following

$$X_i = X_{\mathrm{mrca}(i,j)} + \Delta X_i \text{ and } Y_j = Y_{\mathrm{mrca}(i,j)} + \Delta Y_j$$

where the $\mathrm{mrca}(i,j)$ represents the most common ancestor for species *i* and *j*, $X_{\mathrm{mrca}(i,j)}$ and $Y_{\mathrm{mrca}(i,j)}$ are the traits for $\mathrm{mrca}(i,j)$, and ΔX_i and ΔY_j are the changes in trait value from their most common ancestor to the extant species X_i and Y_j. Then the covariance between X_i and Y_j can be written as

$$cov[X_i, Y_j] = E[\ (X_{mrca(i,j)} + \Delta X_i\ )(Y_{mrca(i,j)} + \Delta Y_j)\ ]$$

$$= E[X_{mrca(i,j)} Y_{mrca(i,j)}] + E[X_{mrca(i,j)} \Delta Y_j] + E[Y_{mrca(i,j)} \Delta X_i] + E[\Delta X_i \Delta Y_j] \qquad \text{(a)}$$

Note that this method was also used in Blomberg et al. [1] where a discrete version of the OU process was applied. Let $t_j = m\Delta t$ be the branch length from the most common ancestor of *i* and *j* to the species *j* at the tips. Then Y_j can be represented as

$$Y_j = (1 - \alpha_y \Delta t)^{m+1} Y_{mrca(i,j)}$$

$$+\sigma_y \sum_{k=0}^{m} (1 - \alpha_y \Delta t)^{m-k} \{B_{y,(n-m+k+1)\Delta t} - B_{y,(n-m+k)\Delta t}\}$$

The expectation, $E[X_{mrca(i,j)} \Delta Y_j]$, is

$$E[X_{mrca(i,j)} \Delta Y_j]$$

$$= E[X_{mrca(i,j)} \{-Y_{mrca(i,j)} + Y_j\}]$$

$$= -E\left[X_{mrca(i,j)}Y_{mrca(i,j)}\right]$$

$$+ E\left[X_{mrca(i,j)}\left\{(1-\alpha_y\Delta t)^{m+1}Y_{mrca(i,j)}\right.\right.$$

$$\left.\left. + \sigma_y\sum_{k=0}^{m}(1-\alpha_y\Delta t)^{m-k}\left[B_{y,(n-m+k+1)\Delta t} - B_{y,(n-m+k)\Delta t}\right]\right\}\right]$$

$$= \left((1-\alpha_y\Delta t)^{m+1} - 1\right)E[X_{mrca(i,j)}Y_{mrca(i,j)}]$$

$$= \left((1-\alpha_y\frac{t_j}{m}\Delta t)^{m+1} - 1\right)E[X_{mrca(i,j)}Y_{mrca(i,j)}]$$

So $E\left[X_{mrca(i,j)}\Delta Y_j\right] = \left((1-\alpha_y\frac{t_j}{m})^{m+1} - 1\right)E[X_{mrca(i,j)}Y_{mrca(i,j)}]$ (b1)

Similarly, we have

$$E\left[Y_{mrca(i,j)}\Delta X_i\right] = \left((1-\alpha_x\frac{t_i}{m})^{m+1} - 1\right)E[X_{mrca(i,j)}Y_{mrca(i,j)}] \quad \text{(b2)}$$

Since trait evolution on different branches is independent, we have for $k = 0,1,\cdots,m$,

$$E\left[\left[B_{x,(n-m+k+1)\Delta t} - B_{x,(n-m+k)\Delta t}\right]\left[B_{y,(n-m+k+1)\Delta t} - B_{y,(n-m+k)\Delta t}\right]\right] = 0.$$

Hence,

$$E\left[\Delta X_i\Delta Y_j\right] = \left((1-\alpha_x\frac{t_i}{m})^{m+1} - 1\right)\left((1-\alpha_y\frac{t_j}{m})^{m+1} - 1\right)E[X_{mrca(i,j)}Y_{mrca(i,j)}]. \quad \text{(b3)}$$

Substituting (b1), (b2), (b3) into (a) and simplifying, we have

$$cov\left[X_i, Y_j\right] = (1-\alpha_x\frac{t_i}{m})^{m+1}(1-\alpha_y\frac{t_j}{m})^{m+1}E[X_{mrca(i,j)}Y_{mrca(i,j)}]. \quad \text{(c)}$$

To derive $E[X_{mrca(i,j)}Y_{mrca(i,j)}]$, as both species *i* and *j* are the same species from the root to their most common ancestor, X_i and Y_j can be regarded as two different traits of the same species. Since by Brownian motion the evolution after speciation is independent, the correlation ρ between the two Brownian motion processes is

non-zero only when the two traits were evolved in the same species. The correlation can thus be defined

$$E\left[\left[B_{x,(j+1)\Delta t} - B_{x,j\Delta t}\right]\left[B_{y,(k+1)\Delta t} - B_{y,k\Delta t}\right]\right] = \begin{cases} \rho\Delta t, & j = k; \\ 0, & j \neq k. \end{cases}$$

Let t_{ij} be the branch length from the root to the most common ancestor of species i and j. t_{ij} can be divided into short intervals: $t_{ij} = l\Delta t$. Now using

$$Y_{mrca(i,j)} = (1 - \alpha_y \Delta t)^{l+1} Y_0 + \sigma_y \sum_{k=0}^{l} (1 - \alpha_y \Delta t)^{l-k} \left\{B_{y,(k+1)\Delta t} - B_{y,k\Delta t}\right\},$$

we have

$$E\left[X_{mrca(i,j)} Y_{mrca(i,j)}\right] =$$

$$\left((1 - \alpha_x \tfrac{t_{ij}}{l})^{l+1}\right)\left((1 - \alpha_y \tfrac{t_{ij}}{l})^{l+1}\right) E[X_0 Y_0] + \rho\sigma_x\sigma_y \frac{1-(1-\alpha_x\frac{t_{ij}}{l})^{l+1}(1-\alpha_y\frac{t_{ij}}{l})^{l+1}}{\alpha_x+\alpha_y-\alpha_x\alpha_y\frac{t_{ij}}{l}}$$

Following [1] and assuming E[$X_0 Y_0$] = 0 (no correlation at the root), equation (c) becomes $cov[X_i, Y_j] = \rho\sigma_x\sigma_y (1 - \alpha_x \frac{t_i}{m})^{m+1} (1 - \alpha_y \frac{t_j}{m})^{m+1} \frac{1-(1-\alpha_x\frac{t_{ij}}{l})^{l+1}(1-\alpha_y\frac{t_{ij}}{l})^{l+1}}{\alpha_x+\alpha_y-\alpha_x\alpha_y\frac{t_{ij}}{l}}$ which converges to $\rho\sigma_x\sigma_y e^{-\alpha_x t_i} e^{-\alpha_y t_j} \frac{1-e^{-(\alpha_x+\alpha_y)t_{ij}}}{\alpha_x+\alpha_y}$ as m, l approach infinity (Δt appraoches to 0).

In this study, I use $t_{ij} = g_{ij}$, $t_i = t_j = 1 - g_{ij}$ and $t_i + t_{ij} = g_{ii} = 1$. Therefore the covariance structure used for data analysis is

$$cov[X_i, Y_j] = \rho\sigma_x\sigma_y e^{-(\alpha_x+\alpha_y)(1-g_{ij})} \frac{1-e^{-(\alpha_x+\alpha_y)g_{ij}}}{\alpha_x+\alpha_y}, i, j = 1,2,\cdots,n.$$

Note that when $X_i = Y_j$, we have $\rho = 1$, $\alpha_x = \alpha_y$ and $\sigma_x = \sigma_y$. Thus the variance-covariance structure agrees with the variance-covariance structure in univariate data analysis. Q.E.D.

References

1. Blomberg SP, Garland T Jr., Ives AR (2003) Testing for phylogenetic signal in comparative data: behavioral traits are more labile. Evolution 57:717-745.
2. Jhwueng DC (2010) Problems in Phylogenetics Comparative Methods. Ph.D. Dissertation, Indiana University USA.
3. Øksendal B (2000) Stochastic differential equations: an introduction with applications. Springer-Verlag New York.
