# Supplementary material for: Assessing the Goodness of Fit of Phylogenetic Comparative Methods: A Meta-Analysis and Simulation Study
Source: PLoS One. 2013 Jun 27;8(6):e67001. doi: 10.1371/journal.pone.0067001 (PMC3694968; doi:10.1371/journal.pone.0067001)
Supplement: Figure S1 — Flow of information through the different phases of a systematic review. (DOCX) [file pone.0067001.s002.docx]

Figure S1


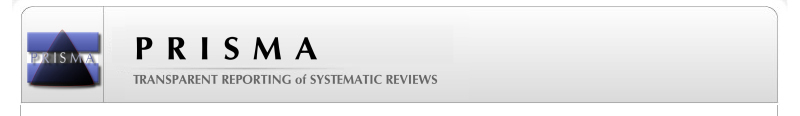
**PRISMA Flow Diagram**

Studies included in quantitative synthesis (meta-analysis)
(n = 43 )

Studies included in qualitative synthesis
(n = 43 )

Full-text articles excluded, with reasons
(n = 0 )

Full-text articles assessed for eligibility
(n = 43 )

Records excluded
(n = 236 )

Records screened
(n = 279 )

Records after duplicates removed
(n = 279 )

Additional records identified through other sources
(n = 0)

## Identification

## Eligibility

## Included

## Screening

Records identified through database searching
(n = 279 )
